# A Case Report of a Rare Hernia: Incarcerated Falciform Ligament Hernia

**DOI:** 10.7759/cureus.37386

**Published:** 2023-04-10

**Authors:** Bilal Koussayer, Timothy Nehila, Sabrina Awshah, Joseph A Sujka, Christopher G DuCoin

**Affiliations:** 1 Surgery, University of South Florida (USF) Health Morsani College of Medicine, Tampa, USA; 2 General, Bariatric, Foregut, Hernia, University of South Florida (USF) Health Tampa General Hospital, Tampa, USA

**Keywords:** internal hernia, robotic surgery, incarcerated hernia, hernia, falciform ligament hernia

## Abstract

Falciform ligament hernias are a rare type of internal hernia that occurs through an abnormal opening in the falciform ligament of the liver. This is the case of a 38-year-old female who presented with a symptomatic enlarging ventral bulge near her umbilicus and was treated with a robotic-assisted laparoscopic falciform hernia repair with mesh. The nonspecific clinical manifestation of a falciform ligament hernia and the low sensitivity of computerized tomography (CT) for these hernias make them hard to diagnose preoperatively. Falciform ligament hernias are mostly attributed to congenital defects, but recently an iatrogenic etiology has also been proposed, given the prior history of laparoscopic surgeries in more recent cases. In our case report, we demonstrate that a robotic-assisted laparoscopic approach is a safe and effective means of correcting this hernia, with an outline of the current literature.

## Introduction

An internal hernia is a protrusion of abdominal viscera through a peritoneal or mesenteric aperture, remaining within the boundaries of the abdominal cavity [1]. A falciform ligament hernia is one rare type of internal hernia that occurs through an abnormal opening in the falciform ligament of the liver, accounting for 0.2% of all internal hernias [1,2]. Presenting signs and symptoms can often be nonspecific, with most case reports describing a patient with an acute onset of abdominal pain and symptoms suggestive of obstruction [2]. Nevertheless, early and accurate management is crucial in preventing morbidity and mortality in these patients. In our report, we demonstrate that a robotic-assisted laparoscopic approach is a safe and effective means of correcting this hernia, with an outline of the current literature.

## Case presentation

**Figure 1:**
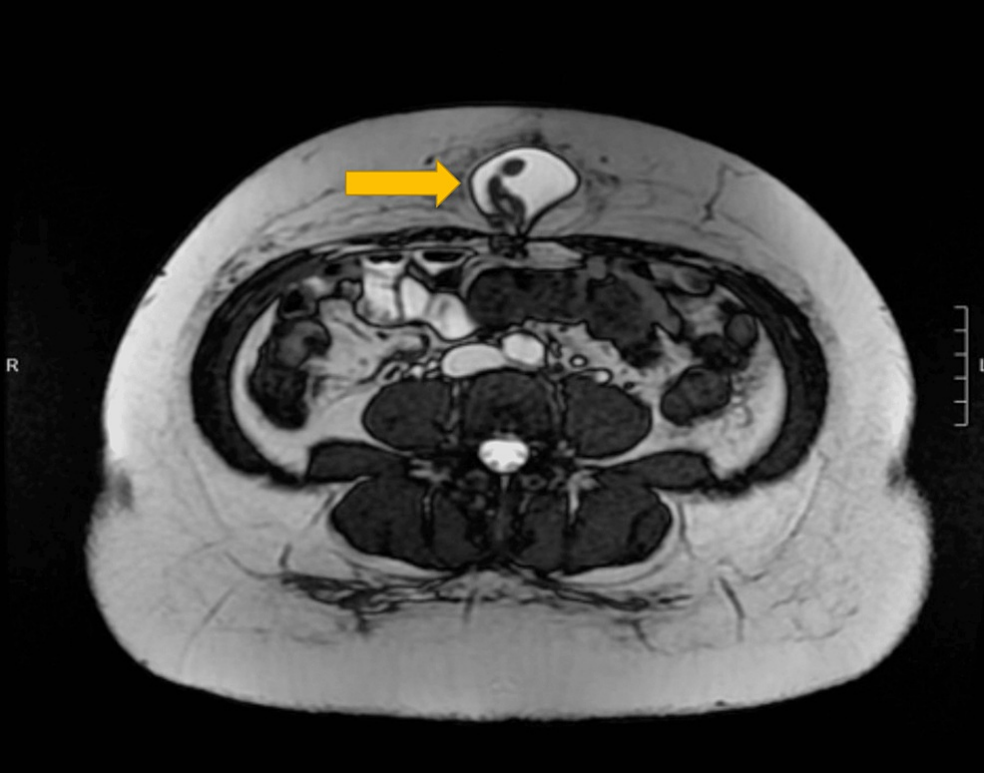
Preoperative MRI MRI with arrow pointing to ventral ligament hernia. The falciform ligament was diagnosed intraoperatively.

This is the case of a 38-year-old female with a past medical history of laparoscopic umbilical herniorrhaphy as a child and uterine fibroids status post-myomectomy. She presented with a symptomatic, enlarging ventral bulge near her umbilicus, not related to her previous surgical incision. She stated that the bulge was tender to palpation and enlarged with activity. Her preoperative workup included magnetic resonance imaging (MRI) to better diagnose soft tissue. This revealed an incarcerated ventral hernia containing only fat (Figure 1). It was decided that the patient would benefit from a robotic-assisted laparoscopic ventral hernia repair with mesh.

The patient was brought to the operating room and prepped, and draped in the normal sterile manner. The abdomen was insufflated with a Veress needle, ports were placed, and the robot was docked. Initially, the hernia was not visible; therefore, the falciform ligament was taken down with electrocautery (Video 1). This allowed us to identify the hernia, which contained an incarcerated falciform ligament and intra-abdominal fat. The hernia was fully reduced (Video 1).

The fascia was closed using a 0-Polydioxanone (PDS) stitch in a figure 8 pattern (Video 1). During the closure, abdominal insufflation pressure was decreased to 8mm Hg. A 9-cm composite circular mesh was selected and sutured in a circumferential pattern using two 2-0 absorbable barbed sutures (Video 1). The patient was discharged the same day, and her postoperative course was unremarkable.

**Video 1:** Robotic Assisted Incarcerated Falciform Ligament Hernia Repair Here we present an intraoperative video of how we treated the falciform ligament hernia using robotic surgery.

## Discussion

Falciform ligament hernias are exceedingly rare hernias that were first identified in the 1930s and have since increased in incidence, with most of the known cases having been diagnosed in the last two decades [2]. As was the case with our patient, the symptoms of a falciform ligament hernia are often nonspecific, and preoperative diagnosis is challenging, with failure to consider an internal herniation and delayed treatment resulting in worse patient outcomes. Of note, one symptom that has been attributed to a falciform ligament hernia is pain relief in the knee-chest position and exacerbation in the supine position [3].

The nonspecific clinical manifestation of falciform ligament hernia suggests an important role in radiological findings in the management of these patients. Anecdotally, the diagnosis of falciform ligament hernia by radiological evaluation of CT imaging has been demonstrated with findings such as clusters of small intestine below the diaphragm surrounding the hepatic falciform ligament, as well as dilated ileal loops between the abdominal wall and liver with the point of stricture at the supposed falciform ligament site [4,5]. However, a review of 37 cases of falciform ligament hernia, published in 2013, found abdominal CT scans to be 35.7% sensitive, suggesting that preoperative imaging may not be a strong diagnostic tool for these patients [6].

While falciform ligament hernias are mostly attributed to congenital defects, an iatrogenic etiology has also been proposed, given the prior history of laparoscopic surgeries in more recent cases [6]. Specifically, laparoscopic cholecystectomy and gastric fundoplication have been implicated in the development of iatrogenic falciform ligament hernias [5]. Although our patient was not status post laparoscopic cholecystectomy or gastric fundoplication, she did have a previous history of multiple abdominal surgeries, which is known to increase the risk for other abdominal hernias [7]. Further, similar histories of non-laparoscopic abdominal surgery have been reported in case reports and systematic reviews of falciform ligament hernia patients [5,8]. Thus, previous abdominal wall surgery history should increase the differential if considering falciform ligament hernias, yet more research is needed to demonstrate an associated risk between the two.

In response to the increasing incidence of falciform ligament hernias in association with the rise in laparoscopic surgical approaches, several risk reduction strategies have been proposed. Internal herniation may be prevented by placing the subxiphoid trocar just to the right of the midline during laparoscopic cholecystectomy [3]. Removal of the subxiphoid port under vision before the desufflation of the pneumoperitoneum can prevent the postoperative chances of herniation [3]. If an aperture is created in the falciform ligament during laparoscopic surgery, division of the falciform ligament, including the round ligament, may prevent future falciform ligament herniation [3].

## Conclusions

Falciform ligament hernia is a rare but increasingly prevalent subtype of internal hernia associated with the modern ubiquity of laparoscopic surgery that must be considered in the differential diagnosis of acute onset of abdominal pain and symptoms suggestive of obstruction. In this case report, we show that robotic-assisted laparoscopy is a safe and effective treatment for falciform ligament hernia.
